# Chicago Medical Society

**Published:** 1910-02

**Authors:** 


### Chicago Medical Society

THE following resolutions were adopted by the Chicago Medical Society, January 11, 1910:

WHEREAS, The Chicago Medical Society is an integral part of a constituent society of the American Medical Association, and therefore vitally interested in the welfare of that great organisation, and

WHEREAS, Certain conditions exist which menace the best interests of the members of the American Medical Association, and of the profession at large;

THEREFORE, *Be It Resolved,* That the Chicago Medical Society in council assembled recommends the following changes in the policies and management of the American Medical Association—namely,

1. The laws should be so amended that no one person will be permitted to hold, at the same time, more than one executive or honorary office in the association.

2. The office of general secretary, and the positions of editor and manager should be separated, and no person should be permitted to fill more than one of these places at one time.

3. The offices of editor and secretary should be filled only by men educated in regular scientific medicine and of unimpeachable record.

4. The number of trustees should be increased.

5. All officers and employes whose duties involve financial responsibility should be bonded.

6. The laws governing admission to membership in the American Medical Association should be so amended as to make it mandatory upon the secretary to enroll applicants who have complied with the provisions of the by-laws governing the same.

7. Space should be set apart in the *Journal* for free and courteous discussion of the policies and methods of the association, or for any other matters which may appeal to the membership at large as bearing upon the interests of the association.

8. Provision should be made for the initiative and referendum.

9. No member should be expelled from the association without a fair trial and full hearing.

10. No person who is a general officer or member of the House of Delegates or Board of Trustees or employee of the American Medical Association shall be eligible to serve as a general officer or member of the House of Delegates, or Council, of any constituent association.

11. *Be It Further Resolved,* That the Secretary of the Chicago Medical Society be instructed to publish these resolutions in full in the bulletin of the society, and to transmit a copy of the same to the *Journal of the American Medical Association* and to the editors of the various state journals.

